# Control and Regulation of Integrated Mitochondrial Function in Metabolic and Transport Networks

**DOI:** 10.3390/ijms10041500

**Published:** 2009-04-01

**Authors:** Sonia Cortassa, Brian O’Rourke, Raimond L. Winslow, Miguel A. Aon

**Affiliations:** 1 Johns Hopkins University, School of Medicine, Division of Cardiology 720 Rutland Ave, 1059 Ross Bldg, Baltimore, MD 21205, USA; E-Mails: bor@jhmi.edu (B.O‘R.); maon1@jhmi.edu (M.A.); 2 Institute for Computational Medicine, 3400 N. Charles St. CSEB 315, Baltimore, MD 21218, USA; E-Mail: rwinslow@jhu.edu (R.W.)

**Keywords:** Mitochondrial computational model, mitochondrial energetics, excitation-contraction coupling, calcium dynamics, metabolic control analysis, distributed control, control by diffuse loops

## Abstract

The pattern of flux and concentration control coefficients in an integrated mitochondrial energetics model is examined by applying a generalized matrix method of control analysis to calculate control coefficients, as well as response coefficients The computational model of Cortassa *et al.* encompasses oxidative phosphorylation, the TCA cycle, and Ca^2+^ dynamics. Control of ATP synthesis, TCA cycle, and ANT fluxes were found to be distributed among various mitochondrial processes. Control is shared by processes associated with ATP/ADP production and transport, as well as by Ca^2+^ dynamics. The calculation also analyzed the control of the concentrations of key regulatory ions and metabolites (Ca^2+^, NADH, ADP). The approach we have used demonstrates how properties of integrated systems may be understood through applications of computational modeling and control analysis.

## 1. Introduction

In the midst of a transition between analytical and integrative periods in biology, cell physiology is moving from the unraveling of most metabolic pathways, along with their constituent enzymes, towards quantification of integrated networks of reactions [1]. In this new direction, Systems Bioenergetics focuses on metabolism, not only as a mesh of biochemical reactions, but also as an evolving, dynamic, and spatially organized, mass-energy-information network [2]. Germane to their functionality is the understanding of how those networks are controlled and regulated as a whole.

Metabolic Control Analysis (MCA) was among the earliest attempts to introduce a generalized quantification method that could be applied regardless of pathway complexity. Independently developed by Kacser and Burns [3] and Heinrich and Rapoport [4], and elaborating on the work of Higgins [5], MCA has been extended and improved (see [6] and [7] for reviews). MCA addresses the question of what controls, and to what extent, the flux through a metabolic pathway at the steady state.

Given a network of processes of any complexity, the rates of the individual processes constituting such network influence, and are influenced by, the rates of the other interacting processes. In order to quantify control at the steady state, a series of coefficients have been introduced. The most frequently reported value is the flux control coefficient, $C_{E_{k}}^{J_{i}}$ :

(1) $$C_{E_{k}}^{J_{i}}=\frac{\frac{\partial J_{i}}{J_{i}}}{\frac{\partial E_{k}}{E_{k}}}$$

with *J_i_* representing the flux of interest, and *E_k_* the activity of process *k,* whose control is quantified by $C_{E_{k}}^{J_{i}}$. This analysis requires the system to be continuous (differentiable) in the neighborhood of a steady state. The flux control coefficient measures how much of the flux through a pathway (e.g. oxygen consumption flux, J_i_) or a single step (e.g. adenine nucleotide translocase, J_i_) would be modified if any activity in the system changes through a modification in either enzyme abundance or activity (e.g., changes in the rate of ATP synthase, E_k_). An analogous definition applies for the metabolite concentration control coefficient, $C_{E_{k}}^{M_{i}}$. These two coefficients reflect *global* properties of the network — both are dependent on the rates of *all processes* in the system. If, for a given process, the magnitude of the control coefficient is close to 1.0, it indicates that a change in the flux (e.g. O_2_ consumption rate) will be almost proportional to the change in the activity of the process under study. Flux control coefficients can be either positive or negative (e.g., an increase in the activity of a negative controlling step will decrease the flux), but the summation theorem requires that the sum of all flux control coefficients at the steady state should equal 1.0 [6].

On the other hand, the elasticity coefficient, ${ɛ}_{Sj}^{v_{k}}$, quantifies the dependence of the rate of a specific process, *k*, on the concentration of an intermediate or effector in the network, *S_J_*. The elasticity coefficient as defined in Equation 2, computes the magnitude by which an enzyme activity (e.g. ATP synthase, v_k_) changes upon variation in the level of a substrate or an effector (e.g. ADP, *S_j_*). In contrast to control coefficients, elasticities depend upon *local* properties of the enzyme, and the concentrations of its substrates and effectors. In practical terms, elasticities correspond to the slope of the relationship between the initial rate of an enzyme-catalyzed reaction and the concentration of the substrate (or an effector):

(2) $${ɛ}_{Sj}^{v_{k}}=\frac{\frac{\partial v_{k}}{v_{k}}}{\frac{\partial S_{J}}{S_{J}}}$$

For a more in depth discussion and development of the analytical tools of MCA, the reader is referred to the book authored by D. Fell [6] and to Cortassa *et al.* [8] for further analysis of the matrix method as applied in the present work.

Rigorously, MCA applies to the analysis of *steady states* raising concerns about its use in time-dependent behavior (see [8] for a more detailed discussion). However, Ingalls and Sauro [9] have reported the validity of applying MCA to averages of time-dependent behavior exhibited by systems undergoing periodic dynamics. It was shown that under those conditions the summation and connectivity theorems of MCA are fulfilled [9].

## 2. Control and Regulation of Oxidative Phosphorylation and ATP Provision in the Heart

Mitochondria were among the first biological systems subjected to MCA, and oxidative phosphorylation was the prime example illustrating how control is distributed among several “ratecontrolling”, rather than “rate-limiting”, steps [10]. This was the expected outcome from a method accounting for coefficients assessing the impact of both systemic and individual steps in the biochemical network. MCA also revealed that the pattern of control changes more quantitatively (control strength) rather than qualitatively (steps involved) following changes in physiological conditions [11–14]. Since its introduction, MCA has been extensively applied to study properties of isolated mitochondria from liver [15–17], muscle [18,19] and other sources [20], although few studies have been carried out using intact cells [11,12] or tissue [8].

The problem of control and regulation of oxidative phosphorylation is of enormous importance in the heart, an organ pumping roughly 75 gallons of blood per hour for about 100 years [21]. Over 90% of heart metabolism is aerobic [22], accounting for nearly 10% of the O_2_ consumption of the body at rest. Mitochondria provide the bulk of the ATP needed for cardiac muscle contraction (about two thirds) and sarcolemmal and sarcoplasmic ion transport (one third), responsible for the electrical activity of the cardiac cell [22]. Thus, in the heart, oxidative phosphorylation represents the dominant source of energy for matching metabolic/contractile demand.

Based on MCA of an integrated model of mitochondrial energetics and Ca^2+^ dynamics, we introduced the notion of “push” and “pull” in the context of control of the respiratory flux [23] and the framework of the top-down method of MCA with NADH as a “hinge” [24]. A “push” condition happens when the steps controlling the respiratory flux occur upstream of NADH (e.g. TCA cycle), whereas “pull” corresponds to a situation in which respiration is mainly controlled by processes downstream of NADH (e.g. adenine nucleotide translocator, ANT, ATPase, respiration itself). In a “push” condition, the NADH/NAD^+^ ratio is low (oxidized redox potential), and respiratory flux depends on activation of the TCA cycle to regenerate NADH. Under these conditions, alphaketoglutarate (KGDH) and isocitrate (IDH) dehydrogenases become the main rate-controlling steps of the respiratory flux. On the contrary, a “pull”-type of control occurs when the control of respiration shifts downstream of NADH (e.g. to ANT and ATPase), associated with a high NADH/NAD^+^ ratio (reduced redox potential). In this context, it is instructive and useful to emphasize the differences in the meaning of the terms control and regulation, a key conceptual advance contributed by MCA [1,7]. The definitions we use here are somewhat different from that proposed in [25]. Under “push” conditions, respiration in mainly *controlled* by KGDH and IDH and *regulated* by Ca^2+^, that is, *control* indicates the influence of the rate of a metabolic (e.g. KGDH) or transport reaction on the flux (e.g. respiration), whereas *regulation* refers to the modulation of an enzyme activity (e.g. KGDH) or pathway in response to the change in the level of a metabolite (e.g. ADP) or ionic species (e.g. Ca^2+^ activation of KGDH or, in MCA terms, positive elasticity of KGDH toward Ca^2+^). Recognizing the difference between control and regulation is not merely semantic; it informs us about what to look for in order to understand network function. More specifically, it is a key issue for recognizing that enzymatic or transport reactions as well as posttranslational modifications *control* whereas metabolic intermediates, ions or second messengers, *regulate*.

## 3. Control and Regulation of Overall Network Function in Isolated Mitochondria

Very recently, we applied a generalized matrix method of control analysis [8,26,27], to calculate flux and concentration control coefficients, as well as response coefficients to an integrated cell model[8] of Excitation-Contraction (EC) coupling and Mitochondrial Energetics (ECME model) [28]. This quantitative analysis revealed the complex interdependence of sarcolemmal, cytoplasmic, and mitochondrial processes that contribute to the control of energy supply and demand in the heart.

**Scheme 1.**
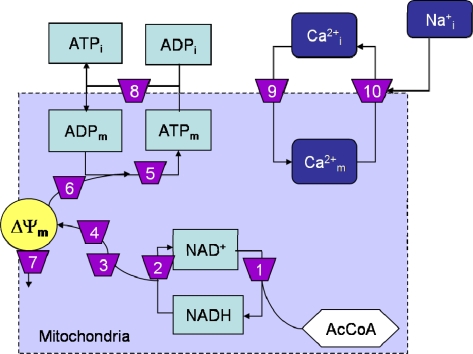
Scheme of the model ECME subjected to control analysis. Processes accounted for by the ME model are numbered according to the following key:

In the present work, we present selected results obtained with the generalized method of control analysis by Reder [27], as applied to the isolated mitochondrial energetics (ME) model (Scheme 1). The matrix method does not assume complete fulfillment of the MCA theorems; however, as expected from applying it to the steady state, we found that the summation theorems for the flux (= 1.0) and intermediate concentration (= 0) control coefficients were satisfied, along with the summation of the response coefficients (= −1.0) for metabolites [8]. All of these tests ascertain the consistency of the calculations performed in accordance with the principles of MCA.

**Figure 1.**
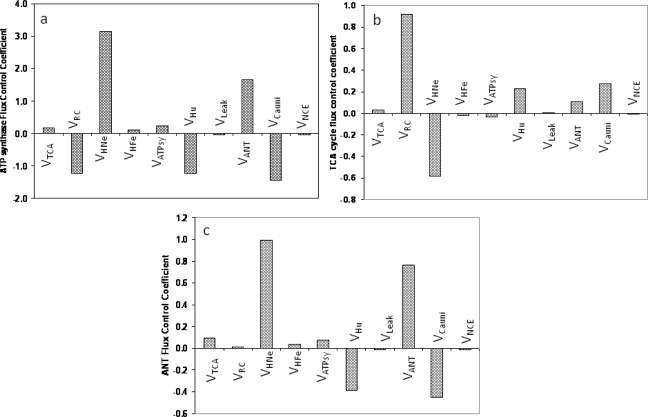
Control of metabolic fluxes in the ME model. The control coefficient by each of the steps of the ME model are represented for (a) the ATP synthase, (b) the TCA cycle, and (c) the ANT. See the legend of Scheme 1 for abbreviations. Panel (a) reprinted from Cortassa, S.; O’Rourke, B.; Winslow, R.L.; Aon, M.A. Control and regulation of mitochondrial energetics in an integrated model of cardiomyocyte function. *Biophysical Journal* **2009**, 96, 2466 – 2478 with permission from Elsevier.

The analysis revealed the highly distributed control exerted by different mitochondrial processes on ATP synthesis, the TCA cycle, and the ANT flux (Figure 1). The ATP synthesis flux, V_ATPsy_, is mainly controlled negatively by the activity of the respiratory chain carriers (V_RC_); positively by the proton transport associated with respiratory electron transfer chain (V_HNe_); and negatively by the proton transport linked to ATP synthesis (V_Hu_). Additionally, V_ATPsy_ is significantly and negatively controlled by the Ca^2+^ uniporter (V_Cauni_) and, positively, by the adenine nucleotide translocator (V_ANT_) (Figure 1a). Relatively minor, but significant, positive control of ATP synthesis is contributed by the TCA cycle (V_TCA_) and the proton flux associated with succinate-driven respiration (V_HFe_), and the ATP synthase (V_ATPsy_).

The control of the flux through the TCA cycle shows some significant differences with respect to ATP synthesis. Among the most relevant is the strong positive control by the activity of the respiratory chain carriers (V_RC_) followed by V_Cauni_ > V_Hu_ > V_ANT_ whereas V_HNe_ exerted a strong negative control (Figure 1b). This control pattern reveals that an increase in the amount of electron carriers in the respiratory chain as well as the H^+^ flux through the ATPase, which consumes the proton motive force (pmf) and activates respiration, exert positive control on the TCA cycle. However, an increase in the pmf will exert a negative control over the TCA flux due to the negative control that V_HNe_ exerts on respiration. That is, any process downstream of NADH that increases respiration, thus consuming NADH, will in turn exert a positive control over the TCA cycle.

The adenine nucleotide translocator (ANT) has been shown to exert a significant control on the respiratory flux in several earlier studies [10,15,29]. Figure 1c depicts how the flux through the ANT (V_ANT_) is also controlled in a highly distributed manner. The V_ANT_ is strongly and positively controlled by V_HNe_ and V_ANT_, whereas V_Hu_ and V_Cauni_ exert negative control (Figure 1C). Relatively lower and positive control on V_ANT_ was exerted by V_TCA_ > V_ATPsy_ > V_HFe_ > V_RC_.

We have previously pointed out the relevance of metabolites such as ATP_i_, an energetic intermediate such as ΔΨ_m_, and an ionic species such as mitochondrial Ca^2+^ (Ca^2+^ _m_) as main regulators of V_O2_ [8]. Due to the importance of mitochondrial matrix redox and phosphorylation potentials as key indicators of mitochondrial physiology, here we focused on the processes controlling the concentrations of NADH and ADP in the mitochondria. As expected, V_TCA_ and V_RC_ are the two main controllers of mitochondrial NADH levels; the concentration control coefficients were almost the same (~ 23%) but with opposite signs: positive for V_TCA_ and negative for V_O2_. About ten-fold lower positive control was exerted by both V_HNe_ and V_Cauni_ on NADH concentration, as compared with V_TCA_ and V_O2_, whereas V_NCE_ > V_Hu_ exerted low magnitude negative control. ADP_m_ was positively controlled by V_Cauni_ > V_Hu_ > V_ANT_ and negatively by V_HNe_ > V_TCA_ ≈ V_ATPsy_ > V_HFe_ > V_RC_.

## 4. Control by Diffuse Loops in Mitochondria

Energy supply and demand in the heart appears to be controlled by *diffuse loops* [8]. The concept of diffuse loops emerged from studies trying to visualize the structure of control of metabolic and transport networks of the myocyte as a whole [8]. We defined control by diffuse loops as the control exerted by a process A over another, e.g., C (mechanistically unrelated or indirectly related to process A) through at least one intermediate process B. We pointed out that the existence of diffuse loops provides a rationale for understanding that an action on one part of the network (e.g. by a pharmacological agent) may bring about changes in other parts without obvious direct mechanistic links between them.

Mitochondria also exhibit control by diffuse loops. The control exerted by some mitochondrial processes on the flux of ATP synthesis (Figure 1) can be readily interpreted based on first principles. Discriminating between the proton fluxes associated with respiratory electron transport (V_HNe_ and V_HFe_), and ATP synthesis (V_Hu_) we show that ATP synthesis is positively controlled by the buildup of the pmf (V_HNe_ and V_HFe_), and negatively controlled by the flux of H^+^ associated with ATP synthesis. The results indicate that when the pmf is built up by V_HNe_, it feeds back positively on the ATPase (i.e., higher ΔΨ_m_, higher ATPase activity) whereas, when the pmf is dissipated (mainly through ΔΨ_m_), the ATPase activity decreases. In the ME model, V_O2_ and V_ATPsy_ depend upon both ΔΨ_m_ and ΔpH [23]. The overall fluxes of respiration [8] and ATP synthesis (Figure 1a) are strongly dependent on ΔΨ_m_ within a certain range, and follow the general flux-force relationship and dependence upon ΔΨ_m_ and ΔpH described for numerous biological free-energy transduction processes [23,30,31]. These effects explain the diffuse loop acting as a negative control exerted by V_CaUni_ on ATP synthesis (Figure 1a). The latter control is mediated by ΔΨ_m_ dissipation due to the electrogenic uptake of Ca^2+^ through the uniporter [23]. This ΔΨ_m_–mediated diffuse loop can be further clarified if we take into account the dual effect of Ca^2+^ transport; on the one hand activating the TCA cycle dehydrogenases thereby stimulating NADH production and respiration, and on the other hand, dissipating ΔΨ_m_ because of the inward transport of positive charges. Quantitatively, the negative control by V_CaUni_ on ATP synthesis happens because ΔΨ_m_ dissipation is larger than the Ca^2+^-mediated TCA cycle activation.

**Figure 2.**
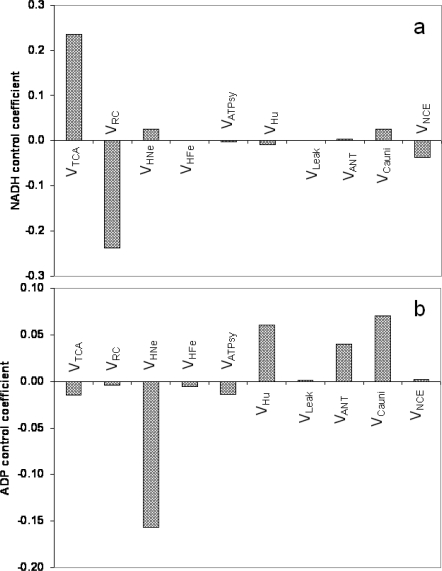
(a) Control of NADH and ADP concentrations in the ME model. Represented are the control coefficients of NADH (a) and ADP (b), extracted from the computed matrix of metabolite concentration control coefficients.

Although less intuitive at first sight, the control over V_ANT_ by numerous mitochondrial processes can be readily understood if we take into account the diffuse loop involving ΔΨ_m_ dissipation. Any mitochondrial process contributing to the buildup of ΔΨ_m_ (e.g. TCA, V_HNe_) will control positively V_ANT_ whereas those dissipating ΔΨ_m_ (e.g. V_CaUni_, V_Hu_) will exert a negative control owing to its electrogenic nature (ATP efflux/ADP influx is equivalent to a negative charge moving out, driven by the electrical gradient). Control of metabolite concentrations also involves diffuse loops. Mitochondrial Ca^2+^, NADH and ADP concentrations are examples (Figure 2).

In the case of Ca^2+^, the interpretation is straightforward, since its concentration is positively controlled by the uniporter (control coefficient = 0.609) and negatively controlled by Na^+^Ca^2+^ exchanger (V_NCE_; control coefficient = −0.606) (not shown). Additionally, in the case of NADH, the major control over its concentration is given, positively, by the TCA cycle, and, negatively, by the activity of the respiratory chain carriers (V_RC_) (Figure 2a). More indirectly, and to a much lesser extent, mitochondrial NADH is controlled positively and negatively, respectively, by V_Cauni_ and V_NCE_ through matrix Ca^2+^. Thus, from a quantitative standpoint, mitochondrial Ca^2+^ levels are controlled by the Ca^2+^ uniporter and the Na^+^Ca^2+^ exchanger, whereas NADH concentration is largely controlled by the opposing activities of the V_TCA_ and V_RC_ and to a lower degree by V_Cauni_ and V_NCE_. A consequence of this analysis is that the TCA cycle or the respiratory chain, and not the Ca^2+^ transporters, will normally predominate in determining the NADH concentration in mitochondria.

As expected, the control of the ADP concentration in the matrix follows an opposite pattern to that of ATP synthesis (compare Figures 1a and 2b). ADP is positively controlled by all the processes that dissipate the pmf such as V_Cauni_ > V_Hu_ > V_ANT_ whereas ADP is negatively controlled by all other processes that tend to recharge the pmf being V_HNe_ the strongest then followed by V_TCA_ > V_ATPsy_ > V_HFe_ > V_RC_ (Figure 2b). V_ANT_ was the only exception to this control pattern since both ADP and the ATP synthesis flux were positively controlled by the ANT although to a much lower extent in the case of ADP concentration.

## 5. Discussion

By applying a generalized matrix method of Metabolic Control Analysis to study the network of metabolic and transport reactions in a model of mitochondrial energetics, we could investigate the control of basic physiological functions such as respiration and ATP synthesis as well as the matrix concentration of key metabolites and ions such as NADH, ADP, and Ca^2+^. This work shows how control by diffuse loops also operates at the mitochondrion level. Diffuse loops were previously found in a similar analysis applied to an integrated model of cardiomyocyte function (ECME model, [8,28]. We defined control by diffuse loops as the influence exerted by a process A over another C through at least one (may be several, see below) intermediate process B [8]. As an example, the negative control exerted by the Ca^2+^ uniporter (process A) over ATP synthesis (process C) is mediated by ΔΨ_m_ (process B). The mechanistic relationship between ΔΨ_m_ and ATP synthesis allows us to see that decreasing ΔΨ_m_ within a certain range (e.g. by Ca^2+^ transport through the uniporter) has a strong negative impact on the ATP synthase activity (see Figures A5B and A6B in [23]).

Another contribution of the present work is to quantify the impact of Ca^2+^ transport on mitochondrial matrix NADH level. An extensive body of work in the literature shows the distinct role of Ca^2+^ on mitochondrial energetics as well as on the contractile and electrical functions of the heart [32,33]. Germane to our discussion is the impact of Ca^2+^ on mitochondrial redox. Our previous work showed that mitochondrial physiology can be characterized by “push” or “pull” conditions depending on the control residing on mitochondrial processes upstream or downstream of NADH. The fact that mitochondria are in “push” or “pull” is largely associated with the NADH/NAD^+^ ratio; with high ratios corresponding to “pull”. Previously reported data in the literature confirm that the NAD^+^/NADH ratio in heart or liver mitochondria is much lower than in the cytosol (8 *versus* 725 [34]) in tune with a more reduced NADH pool [35]. The pattern of control of oxidative phosphorylation is also in agreement with a “pull” rather than a “push” condition in isolated mitochondria [25] as well as in living cells [36] or tissues [37]. These facts highlight the importance of the mitochondrial matrix NADH/NAD^+^ status for the control of mitochondrial function.

Previously, we showed a large control by the respiratory chain and the ANT on the respiratory flux under “pull” conditions [23]. The latter situation corresponds closely to that described by Borutaite *et al.* [38] regarding the control of respiration in heart mitochondria. Moreover, experimental data obtained for isolated rat liver cells show that control of respiration and oxidative phosphorylation is primarily downstream of NADH [36]. Accordingly, rat liver mitochondria would be predominantly in the pull condition, inasmuch as 49% of the control is exerted by the processes of ATP synthesis, transport and consumption, 22% by proton cycling not coupled to ATP synthesis, and only 29% by respiration and upstream processes.

Since processes downstream of NADH are flux-controlling under “pull”, ATP synthesis is less stimulated by cytoplasmic Ca^2+^ when compared to “push” conditions, a condition in which the TCA cycle dehydrogenases are more rate-controlling. A drawback of the “push” condition is that the simulated NADH levels are much lower than the values that have been observed experimentally. Brown *et al.* [36] estimated that 15 – 30% of the control over respiration would be exerted by processes involved in NADH generation. However, in our simulations, under “pull” conditions, the stimulation of oxidative phosphorylation by increasing cytoplasmic Ca^2+^ concentration is small. These results led us to conclude that cytoplasmic Ca^2+^ is better able to stimulate the rate of mitochondrial ATP synthesis only when the TCA cycle exerts a significant control on respiration [23].

In a comprehensive review Brown [25] stated that: “…there is no simple answer to the question “what controls respiration?” Part of the difficulty not only resides in the extreme reactivity of mitochondria to incubation and source conditions, but also stems from the lack of a clear quantification of control and regulation. As stated above, *control* is the extent to which a flux through a pathway, or the concentration of an intermediary metabolite, is altered by changing the activity of one or more steps, and is quantified by *flux* and *concentration control coefficients* [6,7]. *Regulation* refers to how the flux of a pathway is modified through the effect on the rate of an individual step by cellular factors, which may include intermediary metabolite concentrations, the ionic environment, etc., and is quantified by the *response coefficient*. The response coefficient measures the fractional change in flux, e.g. respiration, in response to a fractional change in a parameter P (e.g., an effector such as Ca^2+^) other than enzyme activity. Therefore, regulation implies the response of a pathway to an effector on two levels: *(i)* the extent of *control* exerted on the pathway by the enzyme that is the effector’s target, and *(ii)* the *strength or elasticity* of the effect of P on that enzyme. The response coefficient defined in this manner is the product of control and elasticity coefficients.

According to Chance and Williams [39], increased ATP usage causes increased respiration and ATP synthesis. However, the chain of events leading to the realization of this basic mechanism of respiratory control is not straightforward in complex, integrated, metabolic and transport networks. Cortassa *et al.* (2009) [8] produced the first overall calculation of an integrated mitochondrial energetics and EC coupling model of cardiomyocyte function. One main finding was that when the contractile force is close to its maximum, and the energy consuming pumps are nearly at maximal work during the contraction cycle, the control of the respiratory flux is not only widely distributed among mitochondrial processes but also among the major cytoplasmic ATPases: V_AM-ATP_, V_SERCA_, I_NaK_ and plasmalemmal Ca^2+^ ATPase (V_PMCA_) [8]. While V_AM-ATPase_ exerts a positive control on respiration, the others exhibit a negative control: I_NaK_ > V_SERCA_ > V_PMCA_, because of their important effects on Ca^2+^ dynamics. Moreover, the calculation also showed that the control of respiration by cytoplasmic ATPases differs between resting and working conditions, with the ATPases exerting more control at higher work. Surprisingly, when analyzing the control of the ATP synthesis flux we found that, counter-intuitively, the myofibrillar ATPase, V_AM-ATPase_, exerted a negative (*not* the expected positive) control on ATP synthesis. This was a good example of control by diffuse loops [8]: in this case, the decrease in ATP_i_ brought about by an increase in AM-ATPase caused a decrease in the activity of the SERCA pump, which, in turn, increased the concentration of Ca^2+^ _i_ in the cytoplasm. The increase in Ca^2+^ _i_ concentration resulting from the decrease in SERCA activity produced an increase of V_Cauni_, transporting more Ca^2+^ into the mitochondria (also reflected by a large positive control of AM-ATPase on V_Cauni_). The effect of the Ca^2+^ uniporter to dissipate ΔΨ_m_ overrode the small positive control of the ANT brought about by the increase in ADP produced by the AM-ATPase. The net effect was a decreased flux through the ATP synthase [8], a remarkable counterintuitive result.

The predictions raised by the analysis presented here are, indeed, subjected to the soundness of the underlying model. Thus, the validity of the predictions depends upon a correct description of the individual processes taken into account by the model. To this respect, the mitochondrial energetics model has been extensively validated by its ability to reproduce experimental behavior standing as is [23], or coupled to a model of excitation contraction coupling of cardiomyocyte from guinea pig [28]. Moreover, the analysis herein, as applied to isolated mitochondria or single cells, is not limited to small changes in enzyme activity as could be interpreted from the definitions of the coefficients (see Equations 1 and 2) [40]. Control analysis is essentially derived from the modular behavior of the model components which includes built-in stoichiometric and regulatory interactions accounted for by the stoichiometry- and elasticity-coefficients matrices, respectively.

## 6. Conclusions

Overall, concerning the control and regulation of mitochondrial respiration and ATP synthesis, this work shows the following distinct features: *i)* it is distributed to a different extent among several processes; *ii)* it is exerted by diffuse loops acting through intermediary processes; *iii)* the concentration of key regulatory metabolites (ADP, NADH) and ions (Ca^2+^) are in turn controlled by the major ratecontrolling steps of respiratory and ATP synthesis fluxes. This work demonstrates the insight into the properties of integrated systems that may be gained through applications of computational modeling and control analysis.

## Figures and Tables

**Table:** 

Number	Abbreviation	Name
1	TCA	Tricarboxylic acid cycle
2	V_RC_	Respiratory electron transport
3	HNe	Respiratory chain proton pumping
4	HFe	Succinate-driven proton pumping
5	ATPsy	Mitochondrial ATP synthase
6	Hu	Proton pumping through ATP synthase
7	Leak	Proton leak
8	ANT	Adenine nucleotide translocator
9	Ca_uni_	Mitochondrial Ca^2+^ uniporter
10	V_NCE_	Mitochondrial Na^+^ Ca^2+^ exchanger

The scheme shows mass transformation interactions between the state variables of the mitochondrial energetics (ME) model. In the model the TCA cycle starts from AcCoA, i.e. the common intermediary metabolite derived from sugars and fatty acids degradation, thus, not accounting for the activity of pyruvate dehydrogenase, one of the targets of Ca^2+^ regulation [23]. In the scheme, regulatory interactions were omitted for simplicity (see [8] for more details). State variables are indicated in rectangular (ion or metabolites) while boxes depict a light blue background when the state variables participate in conservation relationships (ATP/ADP, NAD^+^/NADH) or a dark blue background for ionic species. The hexagonal box denotes the input of carbon substrate that corresponds to a parameter in the model. Arrowheads point to the products of the numbered processes, whereas lines without arrowheads indicate inputs to those processes. The TCA cycle was considered as a single step in the stoichiometric matrix; however, for the quantification of the elasticity coefficients of the TCA cycle with respect to the intermediates, the disaggregated individual rate expressions and their dependence with respect to Ca^2+^ _m_, NAD^+^, NADH, ADP_m_, and ATP_m_ were taken into account. The individual elasticities were then added together to compute the overall elasticity of the TCA cycle (see [8] for details). ΔΨ_m_ correspond to the mitochondrial membrane potential.

## References

[1] Aon MA Cortassa S Metabolic dynamics in cells viewed as multilayered, distributed, mass-energy-information networks Encyclopedia of Genetics, Genomics, Proteomics and Bioinformatics Jorde L Little P Dunn M Subramaniam SEds; John Wiley & Sons Inc New York, USA 2006 3

[2] Saks V Dzeja PP Guzun R Aliev MK Vendelin M Terzic A Wallimann T System analysis of cardiac energetics-excitation-contraction coupling: Integration of mitochondrial respiration, phosphotransfer pathways, metabolic pacing, and substrate supply in the heart Molecular System Bioenergetics Energy for Life Saks VEd; Wiley-VCH Verlag GmbH&Co. KGaA Weinheim, Darmstadt, Germany 2007 367 405

[3] Kacser H, Burns JA (1973). The control of flux. Symp. Soc. Exp. Biol.

[4] Heinrich R, Rapoport TA (1974). A linear steady-state treatment of enzymatic chains. General properties, control and effector strength. Eur. J. Biochem.

[5] Higgins J Dynamics and control in cellular reactions Control of Energy Metabolism Chance B Estabrook RW Williamson JREds; Academic Press New York, USA 1965 13 46

[6] Fell DA (1996). Understanding the Control of Metabolism.

[7] Cortassa S, Aon MA, Iglesias AA, Lloyd D (2002). An Introduction to Metabolic and Cellular Engineering.

[8] Cortassa S, O’Rourke B, Winslow RL, Aon MA (2009). Control and regulation of mitochondrial energetics in an integrated model of cardiomyocyte function. Biophys. J.

[9] Ingalls BP, Sauro HM (2003). Sensitivity analysis of stoichiometric networks: An extension of metabolic control analysis to non-steady state trajectories. J. Theor. Biol.

[10] Groen AK, Wanders RJ, Westerhoff HV, van der Meer R, Tager JM (1982). Quantification of the contribution of various steps to the control of mitochondrial respiration. J. Biol. Chem.

[11] Aon MA, Cortassa S (1998). Catabolite repression mutants of Saccharomyces cerevisiae show altered fermentative metabolism as well as cell cycle behavior in glucose-limited chemostat cultures. Biotechnol. Bioeng.

[12] Cortassa S, Aon MA (1997). Distributed control of the glycolytic flux in wild type cells and catabolite repression mutants of Saccharomyces cerevisiae. Enz. Microbial Technol.

[13] Fritzen AJ, Grunnet N, Quistorff B (2007). Flux control analysis of mitochondrial oxidative phosphorylation in rat skeletal muscle: pyruvate and palmitoyl-carnitine as substrates give different control patterns. Eur. J. Appl. Physiol.

[14] Rigoulet M, Mourier A, Devin A, Saks V (2007). Organization and regulation of mitochondrial oxidative phosphorylation. Molecular System Bioenergetics Energy for Life.

[15] Gellerich FN, Bohnensack R, Kunz W (1983). Control of mitochondrial respiration. The contribution of the adenine nucleotide translocator depends on the ATP- and ADP-consuming enzymes. Biochim. Biophys. Acta.

[16] Letellier T, Malgat M, Mazat JP (1993). Control of oxidative phosphorylation in rat muscle mitochondria: implications for mitochondrial myopathies. Biochim. Biophys. Acta.

[17] Ventura B, Genova ML, Bovina C, Formiggini G, Lenaz G (2002). Control of oxidative phosphorylation by Complex I in rat liver mitochondria: implications for aging. Biochim. Biophys. Acta.

[18] Jeneson JA, Westerhoff HV, Kushmerick MJ (2000). A metabolic control analysis of kinetic controls in ATP free energy metabolism in contracting skeletal muscle. Am. J. Physiol. Cell Physiol.

[19] Wisniewski E, Kunz WS, Gellerich FN (1993). Phosphate affects the distribution of flux control among the enzymes of oxidative phosphorylation in rat skeletal muscle mitochondria. J. Biol. Chem.

[20] Rossignol R, Letellier T, Malgat M, Rocher C, Mazat JP (2000). Tissue variation in the control of oxidative phosphorylation: implication for mitochondrial diseases. Biochem. J.

[21] O’Rourke B, Cortassa S, Aon MA (2005). Mitochondrial ion channels: Gatekeepers of life and death. Physiology (Bethesda).

[22] Solaini G, Harris DA (2005). Biochemical dysfunction in heart mitochondria exposed to ischaemia and reperfusion. Biochem. J.

[23] Cortassa S, Aon MA, Marban E, Winslow RL, O’Rourke B (2003). An integrated model of cardiac mitochondrial energy metabolism and calcium dynamics. Biophys. J.

[24] Brown GC, Hafner RP, Brand MD (1990). A ‘top-down’ approach to the determination of control coefficients in metabolic control theory. Eur. J. Biochem.

[25] Brown GC (1992). Control of respiration and ATP synthesis in mammalian mitochondria and cells. Biochem. J.

[26] Ainscow EK, Brand MD (1999). Internal regulation of ATP turnover, glycolysis and oxidative phosphorylation in rat hepatocytes. Eur. J. Biochem.

[27] Reder C (1988). Metabolic control theory: a structural approach. J. Theor. Biol.

[28] Cortassa S, Aon MA, O’Rourke B, Jacques R, Tseng HJ, Marban E, Winslow RL (2006). A computational model integrating electrophysiology, contraction, and mitochondrial bioenergetics in the ventricular myocyte. Biophys. J.

[29] Ciapaite J, Bakker SJ, Diamant M, van Eikenhorst G, Heine RJ, Westerhoff HV, Krab K (2006). Metabolic control of mitochondrial properties by adenine nucleotide translocator determines palmitoyl-CoA effects. Implications for a mechanism linking obesity and type 2 diabetes. Febs. J.

[30] Aon MA, Cortassa S (1997). Dynamic Biological Organization Fundamentals as Applied to Cellular Systems.

[31] Westerhoff HV, van Dam K (1987). Thermodynamics and Control of Biological Free-Energy Transduction.

[32] Bers DM (2001). Excitation-Contraction Coupling and Cardiac Contractile Force.

[33] Maack C, Cortassa S, Aon MA, Ganesan AN, Liu T, O’Rourke B (2006). Elevated cytosolic Na^+^ decreases mitochondrial Ca^2+^ uptake during excitation-contraction coupling and impairs energetic adaptation in cardiac myocytes. Circ. Res.

[34] Halestrap AP, Brosnan JT (2008). From metabolic cycles to compartmentation: Another first for Krebs. Biochem J.

[35] Brandes R, Bers DM (2002). Simultaneous measurements of mitochondrial NADH and Ca^2+^ during increased work in intact rat heart trabeculae. Biophys. J.

[36] Brown GC, Lakin-Thomas PL, Brand MD (1990). Control of respiration and oxidative phosphorylation in isolated rat liver cells. Eur. J. Biochem.

[37] Brandes R, Bers DM (1997). Intracellular Ca^2+^ increases the mitochondrial NADH concentration during elevated work in intact cardiac muscle. Circ. Res.

[38] Borutaite V, Mildaziene V, Brown GC, Brand MD (1995). Control and kinetic analysis of ischemia-damaged heart mitochondria: which parts of the oxidative phosphorylation system are affected by ischemia?. Biochim. Biophys. Acta.

[39] Chance B, Williams GR (1956). The respiratory chain and oxidative phosphorylation. Adv. Enzymol. Relat. Subj. Biochem.

[40] Small JR, Kacser H (1993). Responses of metabolic systems to large changes in enzyme activities and effectors. 2. The linear treatment of branched pathways and metabolite concentrations. Assessment of the general non-linear case. Eur. J. Biochem.

